# Early Life Fecal Microbiota Transplantation in Neonatal Dairy Calves Promotes Growth Performance and Alleviates Inflammation and Oxidative Stress during Weaning

**DOI:** 10.3390/ani11092704

**Published:** 2021-09-15

**Authors:** Fernanda Rosa, Tainara Cristina Michelotti, Benoit St-Pierre, Erminio Trevisi, Johan S. Osorio

**Affiliations:** 1 Department of Dairy and Food Sciences, South Dakota State University, Brookings, SD 57007, USA; Tainara.Michelotti@sdstate.edu; 2 Department of Animal Sciences, South Dakota State University, Brookings, SD 57007, USA; benoit.st-pierre@sdstate.edu; 3 Department of Animal Sciences, Food and Nutrition (DIANA), Faculty of Agriculture, Food and Environmental Science, Università Cattolica del Sacro Cuore, 29122 Piacenza, Italy; erminio.trevisi@unicatt.it

**Keywords:** calves, fecal microbiota transplantation, microbiota

## Abstract

**Simple Summary:**

Neonatal dairy calves born with an immature immune system and gut microbiota are exposed to several stressors they have to overcome early in life. Therefore, we proposed the use of gut microbiota from a pre-screened healthy adult donor adapted to the local pathogen load to perform a fecal microbiota transplantation in neonatal dairy calves. Our results suggest that early life fecal microbiota transplantation in neonatal dairy calves is a relatively safe method, influencing growth and development and possibly alleviating the stress caused by weaning procedures.

**Abstract:**

This study aimed to evaluate the effects of early life fecal microbiota transplantation (FMT) on the health and performance of neonatal dairy calves. The donor was selected based on health and production records and fecal material testing negative for infectious pathogens. Sixteen healthy newborn Holstein calves were randomized to either a baseline nutritional program (CON) or 1×/d inoculations with 25 g of fecal donor material (FMT) mixed in the milk replacer (n = 8/TRT) from 8 to 12 days of age. Blood and fecal samples were collected weekly, and calves were weaned at 7 weeks of age. A TRT × Week interaction was observed in haptoglobin, which was reflected in a positive quadratic effect in FMT calves but not in CON. A trend for a TRT × Week interaction was observed in the liver function biomarker paraoxonase, which resulted in greater paraoxonase in FMT calves than CON at three weeks of age. Fecal microbial community analysis revealed a significant increase in the alpha-diversity between week 1 and week 5 for the FMT calves. These results suggest that early life FMT in neonatal calves has positive effects in mediating the inflammatory response and gut microbial maturation.

## 1. Introduction

The traditional paradigm that the fetus develops within a sterile environment, born bacteria-free, and its first contact with bacteria only occurs after birth has been fundamentally challenged [1]; the presence of microbiota has been detected in newborn human meconium [2], amniotic fluid and in the umbilical cord blood [3], as well as in placental membrane [4,5]. Similar to humans, bacteria has been detected in newborn calf meconium [6] and bovine uterus [7]. Early life neonatal gut colonization can be affected by prenatal factors, including maternal microbiota, delivery mode, and postnatal factors such as colostrum intake and quality as well as host luminal pH, diet, and antibiotic treatment [8].

Factors affecting the extrauterine microbiome colonization of the neonate’s gut can fundamentally challenge neonates and put them at risk for immune and metabolic disorders. It has been demonstrated that cesarean-born infants are deprived of the vaginal microbiota inoculation as they pass through the birth canal, and this can predispose them to long-term health consequences [9]. Similarly, maternal microbiota, peptides, and hormones present in colostrum and milk have invaluable benefits in the development of active immunity in neonates across mammalian species [10,11]. In addition to the microbial colonization after birth, there is gradual maturation of gastrointestinal epithelial cells (GEC) that can be influenced by diet [10], length of gestation, and to some extent, antibiotic usage. Overuse of antibiotics has been closely related to disruption of the normal intestinal microbiota (i.e., dysbiosis), and consequently, linked with diseases in humans [12]. In livestock animals, antibiotic usage has been identified as a cause of antibiotic resistance due to its extensive use at a subtherapeutic level to improve growth and enteric disease prevention [13]. However, in the future, this unintentional selection for bacterial resistance to antibiotics will have significant consequences for animal production, especially during the neonatal period, when the gut and immune systems are going through a maturation process. Therefore, any deviation from a normal gut colonization of commensal microbiota in neonates may pose long-term effects on health and growth performance [14]. Thus, manipulation of gut microbiota by feeding either probiotics or prebiotics has been studied in livestock animals as strategies to improve production and health [15,16].

Fecal microbiota transplantation (FMT), which is the transfer of fecal material from a healthy donor into the gastrointestinal tract (GIT) of a recipient with an enteric condition, is used in human patients [17]. FMT has been highly successful in curing enteric diseases such as irritable bowel syndrome and Crohn’s disease in humans [18,19]. FMT has the capacity to shift microbial taxa and restore gut microbiota in humans [20]. Although several studies have demonstrated the benefits of FMT in different human subjects [19,20,21,22,23,24] and piglets [25,26,27,28], the impact of FMT on gut health of neonatal dairy calves remains to be elucidated. 

From a health standpoint, the host immune system and the commensal bacteria present in the GIT need to be in equilibrium; otherwise, disruption of this balance may cause dysbiosis and could trigger an inflammatory response in the host [29]. However, there is a limited understanding of how microbial communities and their interactions with the host, the neonatal calf, can affect gut health and development during preweaning. Therefore, we hypothesized that neonatal gut inoculation with a healthy adult gut microbiota could improve GIT development and maturation early in life. Thus, the objective of this study was to evaluate the effects of performing FMT in neonatal dairy calves on health, growth performance, and immune status during the neonatal and weaning periods.

## 2. Materials and Methods

### 2.1. Experimental Design

The Institutional Animal Care and Use Committee (IACUC) of the South Dakota State University (SDSU) approved all procedures for this study (protocol#A3958-01). This study comprised of a pre-trial phase and a trial phase. The fecal material from the selected cow donor was used in both phases of the study.

### 2.2. Donor Selection and Fecal Preparation

A group of 5 lactating Holstein cows was pre-selected from the herd at the SDSU Dairy Research and Training facility as potential donors under the following criteria: (1) high milk yield production; (2) multiparous cows over 3 lactations; (3) no clinical history of any disease (e.g., retained placenta, and mastitis) during any lactation (Table S1). Fecal samples were collected from each cow, immediately kept on ice, and sent to the Animal Disease Research and Diagnostic Laboratory at SDSU for analysis of infectious pathogens and other risk factors. Stool testing included aerobic culture, fecal float for ova, and PCR detection for the presence of *Mycobacterium paratuberculosis*, *Salmonella*, and *Cryptosporidium* (Table S1). From this group of pre-selected cows, one cow was negative for all the fecal pathogens measured and had no clinical history of any disease during 5 lactations at SDSU; therefore, this cow was designated as the donor. The donor cow was in mid-lactation (122 days in milk) when fecal material was collected for the study. 

Several trials reported that frozen material for FMT has the same efficacy as a fresh fecal sample [30]. Thus, in this current study, the final fresh fecal material from the donor cow was collected via rectum stimulation and immediately stored at −20 °C until further processing. On the first inoculation day of FMT, fecal material was thawed in a warm (37 °C) water bath as described by other studies [25,30], and subsequent homogenous aliquots were kept at −20 °C for the following 4 consecutive inoculations. 

### 2.3. Pre-Trial Phase

Four healthy newborn Holstein calves from the SDSU Dairy Research and Training facility were randomly selected if they met the following criteria: (1) between 2 and 4 weeks of age; (2) fecal score ≤ 2 [scale from 1–4, 1: well-formed-solid feces; 2: soft, pudding-like; 3: runny, pancake batter; 4: liquid, splatters; [31]]. Calves received one FMT inoculation via milk replacer in the morning feeding, and health records were collected for 5 consecutive days. Calves received 25 g (wet weight) of fecal material mixed with a blender in 2.36 L of milk replacer. The proposed 25 g of fecal material was derived from previous FMT performed in humans [~70.55 kg BW of adult [32]]. On the inoculation day, fecal material was thawed in a warm (37 °C) water bath as described by previous studies [25,30]. After the inoculation day, health checks were measured daily, including fecal score (scale from 1–4), rectal temperature, and respiratory score (scale 1–5, 1: normal; 2: runny nose; 3: heavy breathing; 4: cough moist; 5: cough dry [31]). There was no adverse effect to FMT inoculation on milk replacer intake, calf fecal scores did not increase, and the maximum rectal temperature observed was 38.9 °C (Figure S1). A rectal temperature between 38–39 °C is considered as normal body temperature of a calf. Therefore, there was no need to use anti-inflammatory medication for fever (not > 39.4 °C). Thus, no adverse effects were detected during the pre-trial phase, and we proceeded with the trial phase of this study. 

### 2.4. Trial Phase: Study Design and Fecal Microbiota Inoculations

Sixteen healthy newborn male Holstein calves (n = 8/TRT) from the SDSU Dairy Research and Training facility were used for the trial phase in a completely randomized design (CRD) experiment from birth to 7 weeks of age. Calves were used in the experiment if they fit the following criteria: calving difficulty < 3, single calf, and calf birth weight ≥ 36 kg. Calves were processed within 4 h after birth; calf handling included weighing, vaccination with CALF-GUARD^®^ (Bovine Rota-Coronavirus vaccine, Zoetis, MI, USA), navel disinfection with 7% tincture of iodine solution (First priority, Inc., Elgin, IL, USA), and received 3.8 L of high-quality colostrum (>60 mg/L IgG; by hydrometer). The calves were used in the experiment from 20 February until 25 June 2017. They were housed in individual hutches bedded with straw and had ad libitum access to water and antibiotic-free starter grain (Calf 20 Nonmed^®^; 20% CP, Purina Animal Nutrition, MN) throughout the experiment. Calves were randomly assigned to a control treatment (CON) receiving a conventional feeding rate (568 g/d) [31] of milk replacer reconstituted at 12% solids of a commercial antibiotic-free milk replacer (Herd Maker^®^PB; Land O’Lakes Animal Milk products, MN, USA), while calves subjected to the FMT treatment received the same regimen of milk replacer; however, during 8 to 12 d of age, the morning milk replacer was inoculated with 25 g of fecal material from the donor. On the first inoculation day, fecal material was thawed in a warm (37 °C) water bath, as described by other studies [25,30], and subsequent homogenous aliquots were kept at −20 °C for the following 4 consecutive inoculations. After the 5th FMT inoculation, calves received the same feeding program and management as the control calves. During week 6 of age, all calves received milk replacer only once a day (284 g/d) and were completely weaned at week 7 of age (49 day of age). All animals from both control and FMT groups completed the study protocol without the need for any veterinary intervention and no antibiotic treatment was administered throughout the trial.

### 2.5. Animal Measurements and Sample Collection

Growth performance, including body weight (BW) and withers height (WH), were recorded weekly before morning feeding. Health evaluations including fecal score [scale 1–4], rectal temperature, and respiratory score [scale 1–5] were recorded daily throughout the experiment. Individual intakes of milk replacer, starter grain, and water were measured and recorded daily throughout the trial. Blood samples were collected prior to morning feeding from the jugular vein using 20-gauge BD vacutainer needles (Becton Dickinson, Franklin Lakes, NJ, USA). Samples were collected into evacuated tubes (10 mL, BD Vacutainer^®^) containing either serum clot activator or lithium heparin. The first blood sample was collected at 24 h after birth (i.e., 0 week), and subsequent samples were taken weekly until 7 weeks. After blood collection, tubes with lithium heparin were placed on ice (4 °C), and tubes with clot activator were kept at 21 °C (~30 min) until centrifugation. Serum and plasma were obtained by centrifugation of clot activator and lithium heparin tubes, respectively, at 1300× *g* for 15 min at 4 °C. Aliquots of serum and plasma were frozen (−80 °C) until further analysis. Blood samples were analyzed for energy metabolites [i.e., glucose, β-hydroxybutyric acid (BHB)], muscle mass catabolism (i.e., urea and creatinine), liver function [i.e., albumin, cholesterol, total bilirubin, gamma-glutamyl transferase (GGT), paraoxonase (PON), glutamic-oxaloacetic transaminase (GOT)], inflammation [i.e., haptoglobin, ceruloplasmin, Interleukin (IL)-6, and IL-1β], and oxidative stress [i.e., reactive oxygen metabolites (ROM), ferric reducing antioxidant power (FRAP)] using kits purchased from Instrumentation Laboratory (Lexington, MA, USA) following the procedures described previously [33,34,35]. Prior to the morning feeding, weekly fresh fecal samples (~2 g) were collected through rectal stimulation into sterile cryogenic vials (Corning^®^, DNase-RNase free, Cat#337421). Fecal samples were immediately flash-frozen in liquid nitrogen and kept at −80 °C until further analysis.

### 2.6. Polymorphonuclear Leukocytes (PMNL) Isolation

The PMNL were isolated based on procedures described by Rosa, et al. [36]. Briefly, blood samples (~100 mL) were collected from the jugular vein prior to morning feeding at 0, 1, 2, and 3 weeks of age using a scalp vein butterfly (Excel International, CAT# 26702) into evacuated tubes containing ACD solution A (BD, Cat# 268426) and mixed well by inversion and placed on ice until isolation. Samples were centrifuged at 600× *g* for 30 min at 4 °C. The plasma, buffy coat, and ~ one-third of the red blood cells were discarded. The remaining sample was poured into a 50-mL conical tube (Fisher Scientific, Pittsburgh, PA, USA). Twenty-five milliliters of deionized water at 4 °C was added to lyse the red blood cells (RBC), followed by the addition of 5 mL of 5× PBS at 4 °C to restore an iso-osmotic environment. Samples were centrifuged at 900× *g* for 10 min at 4 °C, the supernatants were decanted, and the cell pellet was washed twice with 10 mL of 1 × PBS at 4 °C. The cell suspension was centrifuged at 900× *g* for 5 min at 4 °C, and the supernatants were discarded. The remaining RBC were lysed with 8 mL of ice-cold deionized water, homogenized gently by inversion and 2 mL of 5 × PBS at 4 °C was added. The samples were centrifuged at 900× *g* for 5 min at 4 °C, and the supernatant was discarded. Subsequently, the samples were washed twice using 10 mL of 1× PBS at 4 °C were performed, followed by centrifugation 900× *g* for 5 min at 4 °C. The PMNL pellet was homogenized with 1.5 mL of 1 × PBS at 4 °C, transferred to a 2 mL RNase-DNase-free microcentrifuge tube and centrifuged at 6000× *g* (Sorvall Legend Microcentrifuge 21R) for 5 min at 4 °C. The final PMNL pellet was homogenized in 1 mL of Trizol reagent (Ambion, Carlsbad, CA, USA) and stored at −80 °C until further gene expression analysis. During the PMNL isolation process, an aliquot containing at least 500,000 cells [counted via an automated cell counter (Countess II FL Cell counter from Life Technologies)] was used for PMNL quantification and viability. The PMNL aliquots were incubated for 30 min at 4 °C with a primary antibody for granulocytes (CH138A, Veterinary Microbiology, and Pathology, Washington State University) followed by another 30 min incubation at 4 °C with a secondary antibody (Goat Anti-Mouse IgM, human ads-PE, Southern Biotech). Cells were fixed with 150 µL of 4% paraformaldehyde (Sigma-Aldrich, St. Louis, MO, USA) and preserved at 4 °C until analysis. Flow cytometry data were acquired with the Attune NxT cytometer (Attune^®^ NxT Flow Cytometer, Invitrogen), and the panel with the cell population of interest was analyzed using the Attune NxT cytometer software. 

### 2.7. Fecal DNA Isolation and Bioinformatic Analysis for 16S rRNA Gene-Based Composition Analysis

Fecal DNA was isolated using the QIAmp Fast DNA Stool Mini Kit (Qiagen, Hilden, Germany) with modifications. Briefly, 180–220 mg of stool were weighed in a 2-mL RNase-free O-ring tube containing one stainless steel bead, 5 mm (Qiagen, Hilden, Germany) and placed on ice. For each sample, 1 mL of buffer InhibitEX was added and then homogenized in a Beadbeater (BioSpec Products, Bartlesville, OK, USA) for 30 s. Following homogenization, samples were kept on ice for 1 min, then centrifuged at 20,000× *g* for 1 min at 20 °C. After centrifugation, 600 μL of supernatant were transferred to a new microcentrifuge tube that contained 25 μL of Qiagen Proteinase K, followed by the addition of 600 μL of Buffer AL. The mixture was vortexed for 15 s, then incubated at 70 °C for 10 min in a heat block. After incubation, 600 μL of 96% molecular ethanol was added and vortexed. The mixture was transferred into a QIAamp mini spin column, and the subsequent steps were performed according to manufacturer’s procedures (Qiagen, Hilden, Germany). The DNA quantity and purity were measured using a Nanodrop spectrophotometer (ND 1000, Nanodrop Technologies, Inc., Wilmington, DE, USA).

Unless specified, datasets were analyzed using custom written Perl scripts (available upon request) [37,38]. MiSeq 2 × 300 paired-end reads from 16S rRNA gene amplicons were generated by the University of Minnesota Genomics Center. Overlapping raw forward and reverse reads from the same flow cell clusters were assembled into contigs using the ‘make.contigs’ command from MOTHUR (v 1.44) [39]. Assembled 16S rRNA V1–V3 contig sequences were then screened to meet the following criteria: presence of both intact 27F (forward) and 519R (reverse) primer nucleotide sequences, length between 400 and 580 nt, and an average Phred quality score of Q33 or greater.

Following quality screens, sequence reads were aligned, then clustered into Operational Taxonomic Units (OTUs) at a genetic distance cutoff of 4% sequence dissimilarity. While 3% is the most commonly used clustering cutoff for 16S rRNA, it was originally recommended for full length sequences, and may not be suitable for the analysis of specific sub-regions since nucleotide sequence variability is not constant across the entire length of the 16S rRNA gene. In this context, if 3% is a commonly accepted clustering cutoff for V4 or V4–V5 regions, which are the least variable of the hypervariable regions, then a higher cutoff should be used for the V1–V3 region, since V1 is the most variable region of the 16S rRNA gene [40,41]. 

OTUs were screened for DNA sequence artifacts using the following methods. Chimeric sequences were first identified with the ‘chimera.uchime’ and ‘chimera.slayer’ commands from the MOTHUR open source software package [39,42]. Secondly, the integrity of the 5′ and 3′ ends of OTUs was evaluated using a database alignment search-based approach; when compared to their closest match of equal or longer sequence length from the NCBI ‘nt’ database, as determined by BLAST (Basic Local Alignment Search Tool) [43], OTUs with more than five nucleotides missing from the 5′ or 3′ end of their respective alignments were discarded as artifacts. OTUs with only one or two assigned reads were subjected to an additional screen, where only sequences that had a perfect or near perfect match to a sequence in the NCBI (National Center for Biotechnology Information) ‘nt’ database were kept for analysis, i.e., that the alignment had to span the entire sequence of the OTU, and a maximum of 1% of dissimilar nucleotides was tolerated. 

After removal of sequence chimeras and artifacts, RDP Classifier (Ribosomal Database Project) [44] and BLAST [43] were used for taxonomic assignment of valid OTUs. The List of Prokaryotic Names with Standing in Nomenclature (LPSN) was also consulted for information on valid species belonging to taxa of interest [45].

Alpha diversity indices (Observed OTUs, Chao, and Shannon) were determined using the ‘summary.single’ command from MOTHUR (version 1.44.1) [39] on a dataset subsampled to 5000 reads for each sample. Principle Coordinate Analysis (PCoA) for beta diversity was performed using the same rarefied dataset, by determining Bray–Curtis distances with the ‘summary.shared’ command followed by the ‘pcoa’ command in MOTHUR (version 1.44.1) [39].

### 2.8. RNA Isolation, cDNA Synthesis, and Quantitative PCR (qPCR)

Total RNA was extracted from blood PMNL using Trizol (Invitrogen, Cat# 15-596-026) reagent in combination with the RNeasy^®^ Plus Mini Kit (Qiagen, Cat#74134), following the manufacturer’s instructions with some modifications. Briefly, the cell pellet immersed in TRIzol was transferred to a 2-mL RNase-DNase-free O-ring tube, containing one stainless steel bead, 5 mm (Qiagen, Cat#69989) and homogenized in a Beadbeater (BioSpec Products, Cat#BSP74540) for 30 s. After homogenization, the lysate was transferred to a 2-mL RNase-DNase-free microtube and 200 µL of Phenol: chloroform (Invitrogen™, Cat#AM9730) at 4 °C was added in order to isolate the RNA from the organic phase. After centrifugation at 13,000× *g* for 15 min at 4 °C, the upper phase supernatant was transferred into a new 2-mL RNase-DNase-free microtube. The total RNA was purified using the RNeasy^®^ Plus Mini Kit, and eluted in 50 µL of RNase-free water. The RNA quantity (111.13 ± 102.23 ng/µL; mean ± SD) and purity as 260/280 ratio (1.85 ± 0.15) were determined using Nanodrop. 

The complementary DNA (cDNA) synthesis was performed according to Bionaz, et al. [46]. The cDNA was then diluted 1:4 with RNase-DNase-free water (HyClone, UltraPure™, Cat#10977015). The qPCR was performed in a MicroAmp Optical 384-Well Reaction Plate (Applied Biosystems, Grand Island, NY). Within each well, 4 µL of diluted cDNA combined with 6 µL of a mixture composed of 5 µL 1 × SYBR Green master mix (Applied Biosystems, Woolston Warrington, UK), 0.4 µL each of 10 µM forward and reverse primers, and 0.2 µL of DNase/RNase-free water. Three replicates were run for each sample, and a non-template control was run for each gene analyzed. The qPCR reaction was conducted with a QuantStudio™ 6 Flex Real-Time PCR System (Applied Biosystems) under these conditions: 2 min at 50 °C, 10 min at 95 °C, and 40 cycles with 15 s at 95 °C followed by 1 min at 60 °C. A dissociation curve was performed (gradient from 95 °C to 60 °C to 95 °C for 15 s) to check for amplicon quality.

For this study, relative mRNA expression of each gene was calculated based on a six-point relative standard curve and the internal control genes (ICG) used were golgin subfamily A, member 5 (*GOLGA5*), oxysterol-binding protein-like 2 (*OSBPL2*), and single-strand-selective monofunctional uracil-DNA glycosylase 1 (*SMUG1*), which have been previously used to normalize PMNL gene expression data [47]. The geometric mean of the ICG was used to normalize the expression of the target genes. The stability of the ICG was assessed using the geNorm software [48] with a favorable final pairwise variation of 0.20. The target genes measured within the PMNL samples were associated with pro-inflammatory cascade [Z-DNA binding protein 1 (*ZBP1*), nuclear factor-kappa B subunit 1 (NFKB1), and signal transducer and activator of transcription 3 (*STAT3*)], cell adhesion [L-selectin (*SELL*)], pathogen recognition [toll-like receptor 2 (*TLR2*) and toll-like receptor 4 (*TLR4*)], and pro-inflammatory cytokines [interleukin 1β (*IL1B*) and interleukin 8 (*IL8*)].

### 2.9. Statistical Analysis

Data were analyzed using the PROC MIXED procedure of SAS 9.4 (SAS Institute, Inc., Cary, NC, USA). The model included treatment (TRT; CON and FMT), time (day or week), and their interaction as fixed effects, and calf as a random effect. The autoregressive (1) covariance structure was used for repeated measures for all parameters analyzed. Blood metabolites and gene expression results were log-scale transformed if needed in order to comply with a normal distribution of residuals. Baseline data taken at 24 h after colostrum intake or 0 week were used as a covariate in the model if they were significant (*p* < 0.05). Birth BW and WH were used as a covariate in the model for BW and WH parameters, respectively. A priori orthogonal contrasts were used to determine linear and quadratic effects over time whenever at least a trend (*p* ≤ 0.10) for a treatment × week (TRT × Week) was observed. Statistical significance was declared at *p* ≤ 0.05 and tendencies at *p* ≤ 0.10. Comparisons of alpha diversity indices as well as abundance for bacterial taxonomic groups and OTUs amongst different sample groups treatments were performed in R (Version R-3.6.2) using the non-parametric test Kruskal–Wallis (command ‘kruskal.test’), followed by the Wilcoxon test (command ‘pairwise.wilcox.test’) for multiple pairwise comparisons, which included the Benjamini-Hochberg correction to control for false discovery rate. Statistical significance was set at *p* ≤ 0.05.

## 3. Results

### 3.1. Growth Performance and Health

**Figure 1:**
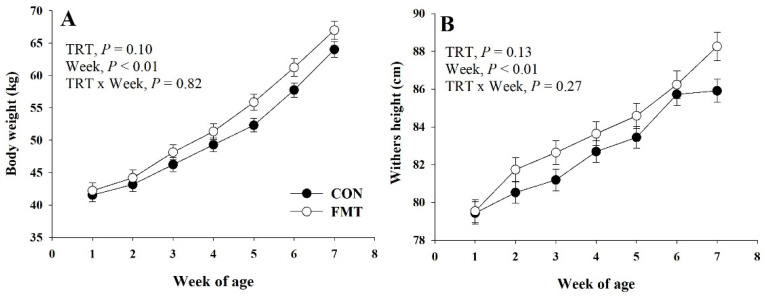
Body weight (**A**) and withers height (**B**) from birth (week 1 of age) to weaning (7 weeks of age) of Holstein dairy calves raised under a conventional nutritional program (CON) or subjected to fecal microbiota transplantation (FMT). The *p*-values for main effects of treatment (TRT) and week, and their interaction (TRT × Week) are shown in each plot. Values are means with standard errors represented by vertical bars.

There was a trend (*p* = 0.10) for greater BW (50.6 vs. 52.8 kg) in FMT calves in comparison to CON (Figure 1). This effect of greater BW in FMT calves than CON was mainly observed from birth until pre-weaning (*p* = 0.06; Table 1) at 6 weeks of age, while this effect seemed to be lessened (*p* = 0.12) at weaning at 7 weeks. A greater (*p* = 0.03) WH in FMT calves in comparison to CON was observed at weaning at 7 weeks (Table 1). Starter intake, total intake, and average daily gain (ADG) were not affected (*p* ≥ 0.86) by treatment effects (Table 1). Gain-to-feed ratio was not affected (*p* = 0.96) by dietary treatments. Neither fecal scores nor rectal temperature were affected (*p* ≥ 0.25) by FMT inoculation (Table 1). This effect was also consistent with a lack of treatment effect (*p* = 0.48) on scour days (fecal score > 3) between FMT calves and CON (Table 1). 

### 3.2. Bacterial Community Composition of the Fecal Donor

**Figure 2:**
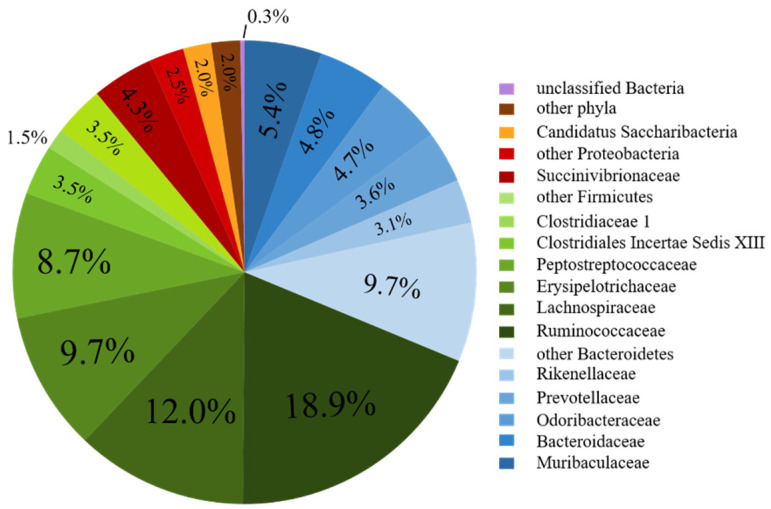
Fecal bacterial composition of the donor used in this study. Abundance is presented as a percentage (%) of the total number of analyzed reads per sample.

A total of 15,141 high-quality sequence reads were used for analysis of the fecal bacterial communities of the donor. Members of the phyla Firmicutes and Bacteroidetes were the most highly represented, combining for 89.1% of sequences (Figure 2). A total of 780 species-level OTUs were identified, including 18 OTUs with a relative abundance greater than 1% that together represented 46.6% of the donor 16S rRNA gene sequences analyzed (Table S2). At least 10 of the main OTUs were likely to correspond to currently uncharacterized bacterial species, as their nucleotide sequence identity to their respective closest valid relatives was less than 95%. The vast majority of these OTUs were found to have a close match to 16S rRNA genes sequences from ‘uncultured’ bacterial species, indicating that they had previously been identified from other studies.

### 3.3. Fecal Microbiota Composition of the FMT Recipients

A total of 580,788 high-quality sequence reads were used for analysis of fecal bacterial communities. Taxonomic composition analysis from both control and FMT groups is shown in Table 2. Across all fecal samples, the two most abundant phyla were Firmicutes and Bacteroidetes (Table 2), while Proteobacteria was highly abundant across calf samples but with a relatively low abundance in the donor feces (6.72%). Eight taxonomic groups were found to vary across calf samples (Table 2; *p* < 0.05), but we were unable to resolve differentially represented groups using pairwise comparisons. Among these, Veillonellaceae, Pasteurellaceae, Enterobacteriaceae, and Actinobacteria had numerically lowest abundance in the feces of the FMT group at week 5.

**Figure 3:**
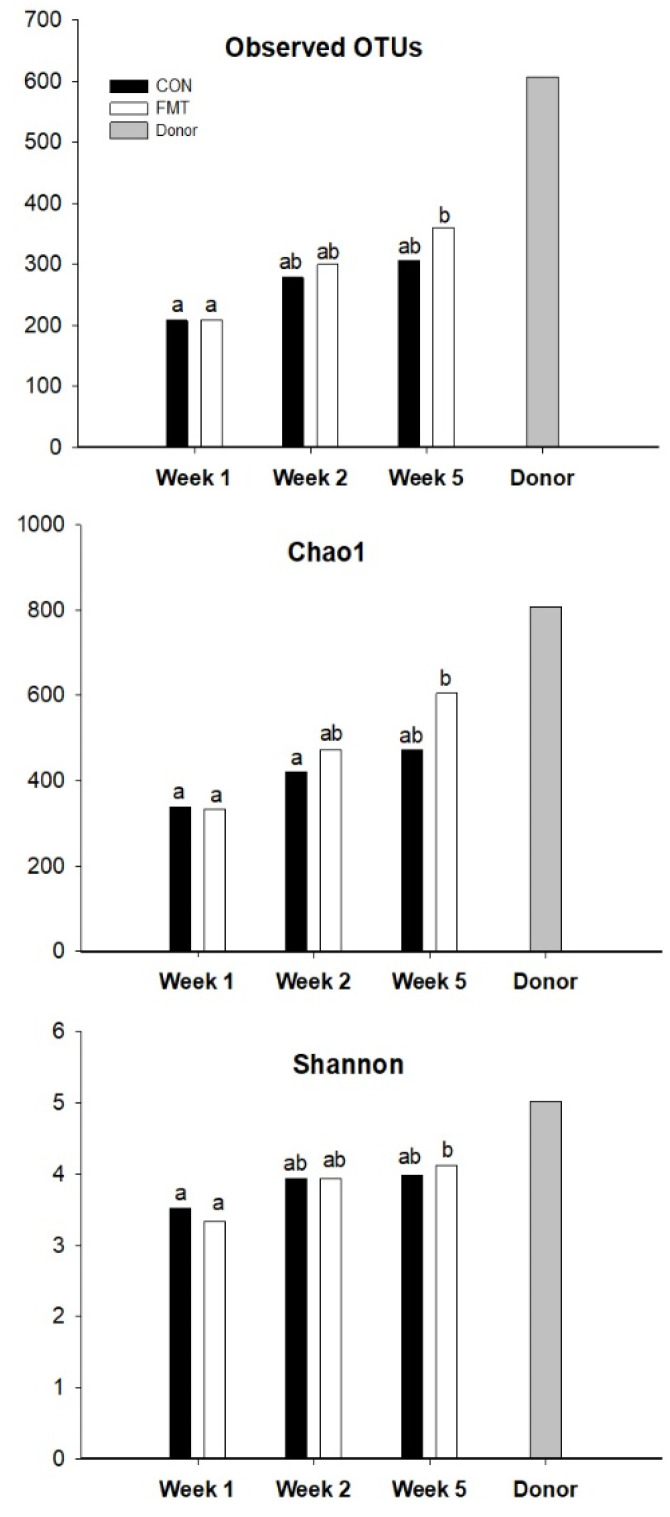
Mean values for alpha diversity indices across groups investigated in this study and donor sample. Alpha diversity indices that show a statistically significant difference (*p* < 0.05) across means are based on the Kruskal-Wallis rank sum test. This analysis did not include the Donor because it was a single sample. ^ab^ Different superscripts indicate that groups are significantly different by the Wilcoxon pairwise test. Chao1 = estimation of the OTUs abundance in samples. Shannon = indicates the diversity of the OTUs in samples.

A total of 3267 species-level OTUs were identified. Analysis of alpha indices indicated a significant increase in diversity between week 1 and 5 for the FMT calves (Figure 3). No clear separation of groups into clusters by PCoA was observed (Figure S2), indicating that samples had overlapping bacterial compositions across different groups. Twelve abundant OTUs were found to vary across calf samples (Table S3; *p* < 0.05), but we were unable to resolve differentially represented groups using pairwise comparisons. Among these, OTUs Bt-01163 and Bt-01238 had a numerically lower abundance in the FMT group in comparison to the CON at week 5, while Bt-01193 and Bt-01356 abundance were numerically higher in FMT calves than CON at week 5. Based on their nucleotide sequence identity to their respective closest valid relatives (82.23–92.92%), at least nine of the main OTUs were likely to correspond to currently uncharacterized bacterial species (Table S3).

### 3.4. Blood Biomarkers

**Figure 4:**
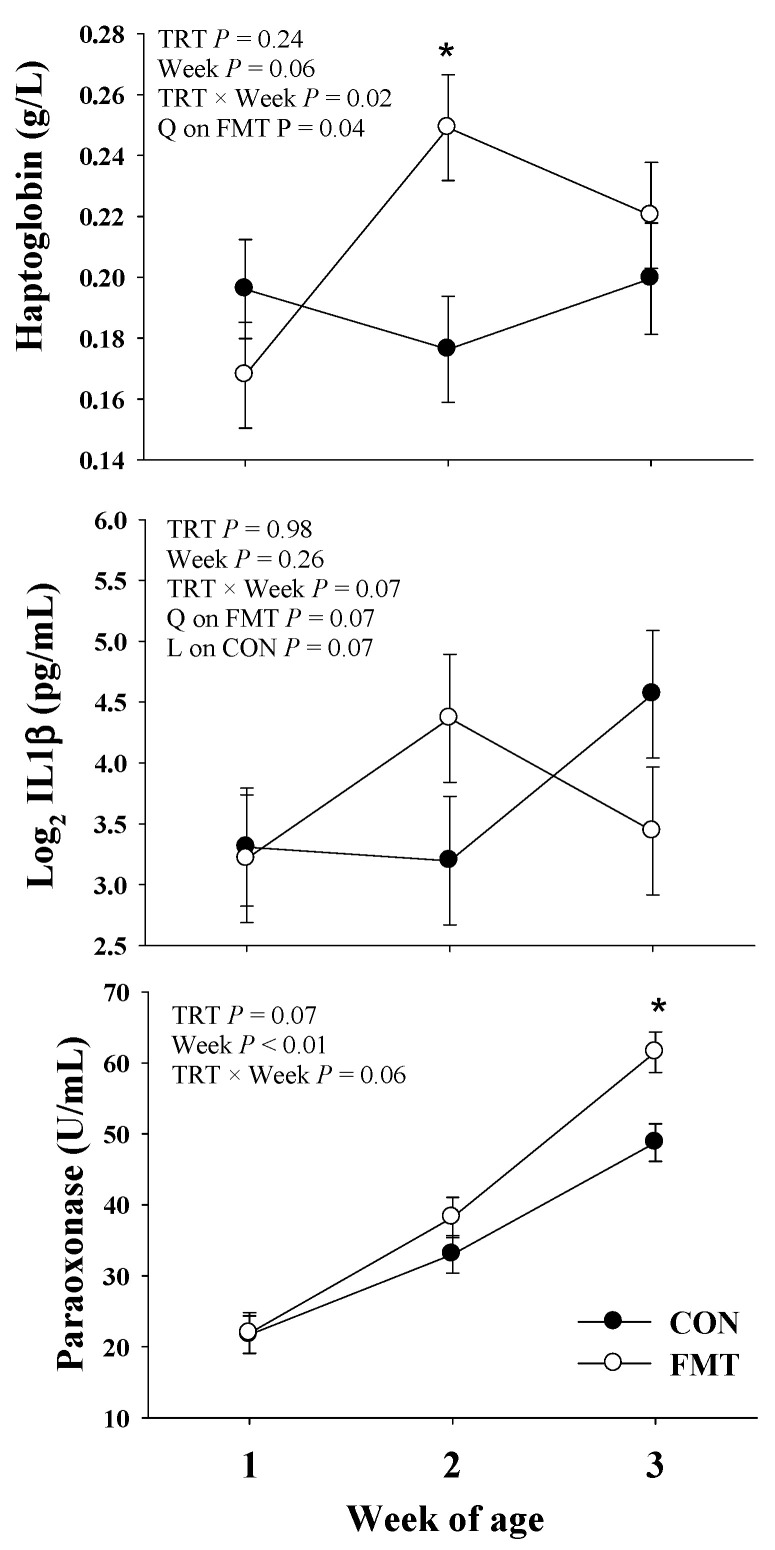
Inflammatory (Haptoglobin and interleukin 1 beta [IL1-β]) and liver function biomarkers (paraoxonase) during the neonatal period in Holstein dairy calves raised under a conventional nutritional program (CON) or subjected to fecal microbiota transplantation (FMT). The *p*-values for main effects of treatment (TRT) and week, and their interaction (TRT × Week) are shown in each plot. The TRT × Week effect (*p* ≤ 0.10) were further analyzed through orthogonal contrast in order to determine linear (L) and quadratic (Q) effects. A significant difference (*p* < 0.05) between treatments in a specific time point was denoted by an asterisk (*). Values are means, with standard errors represented by vertical bars.

The effects of FMT on the host-metabolism during the neonatal and weaning periods were assessed through blood biomarkers of energy metabolism, inflammation, liver function, and oxidative stress. The main effects of treatment, time, and their interactions are presented in Figure 4, and Table 3 and Table 4. 

#### 3.4.1. Energy Metabolism

The blood concentrations of glucose, BHBA, and creatinine were not affected (*p* ≥ 0.20) by treatment in both neonatal and weaning periods (Table 3 and Table 4, respectively). During the neonatal period, a trend (*p* = 0.07) was observed for a TRT × Week (T × W) in urea. However, this effect was mainly associated with transient changes over time rather than actual differences between treatments at a given time point (data not shown). Urea during weaning was lower (*p* = 0.05) in the FMT group in comparison to CON. 

#### 3.4.2. Inflammation

During the neonatal period, there was a T × W (*p* = 0.02) interaction for haptoglobin, and this was reflected by a positive quadratic (*p* = 0.04) effect observed on FMT treatment only (Figure 4). This effect was confirmed by a greater (*p* = 0.01) haptoglobin concentration in FMT calves than CON at 2 weeks of age. Similarly, a trend (*p* = 0.07) for a T × W was observed for IL-1β in blood (Figure 4), which was reflected in a trend (*p* = 0.07) for a positive quadratic effect in FMT calves only, while IL-1β in CON tended (*p* = 0.07) to have a linear increase during the 3 weeks of age (Figure 4). During the weaning period, the inflammatory cytokines IL-6 and IL-1β were lower (*p* ≤ 0.05) in the blood of FMT calves when compared to CON calves (Table 4).

#### 3.4.3. Liver Function

During the neonatal stage, there was a tendency (*p* = 0.06) for a T × W in paraoxonase (Table 3). This effect was explained by greater (*p* < 0.01) paraoxonase in FMT calves than CON at 3 weeks of age (Figure 4). During the weaning period, a T × W (*p* ≤ 0.02) interaction was observed in GOT and GGT. The T × W effect on GOT was mainly attributed to a decline of GOT in CON calves, while GOT remained unchanged in FMT calves from 6 to 7 weeks. The T × W effect on GGT corresponded to a lower (*p* = 0.03) GGT concentration in FMT calves than CON at week 6, which partially explains the trend (*p* = 0.07) for lower overall GGT in FMT calves than CON during the weaning period. 

#### 3.4.4. Oxidative Stress

During the neonatal period, no effects (*p* ≥ 0.20) were observed for ROM and FRAP (Table 3). However, during the weaning period, a T × W (*p* = 0.01) effect was observed for ROM and FRAP. The T × W in ROM was attributed to an increase (*p* < 0.01) in ROM concentration in CON calves from 6 to 7 weeks, while a decrease (*p* = 0.04) occurred in FMT calves from 6 to 7 weeks. The T × W in FRAP was reflected in a decrease (*p* < 0.01) in FRAP in CON calves from 6 to 7 weeks, while FRAP remained unchanged in FMT calves (Table 4).

### 3.5. PMNL Gene Expression

Overall, FMT did not affect PMNL gene expression with the exception of *ZBP1*, where an upregulation (*p* = 0.03) of *ZBP1* was observed in FMT calves compared to CON (Table 5).

## 4. Discussion

We reported for the first time the use of FMT from adult cows in neonatal dairy calves. In either the pre-trial or trial phase, the procedure proved to be safe for neonates with no adverse effects such as milk refusal, increased rectal temperature, or increased respiratory score in FMT animals. These results are in agreement with other FMT studies in swine and companion animals [49,50]. Regarding gastrointestinal health, no severe diarrhea effects were observed during the pre-trial or trial phase. The overall similar fecal scores between FMT and CON coupled with a lack of increase in fecal score post-inoculation suggest that FMT can be a safe procedure. However, further studies are needed to confirm this undetectable effect on fecal scores.

The trend for greater BW coupled with the greater final WH observed in the treated calves suggest that FMT can promote a greater growth performance. We speculate that a distinct set of FMT-mediated gut microbes and metabolites derived from the host-FMT interaction might be associated with improving growth performance in the recipient calves. Similar to these findings in growth performance, Kim, et al. [51] reported an increase in the overall body mass of beef calves at 12 month-old that received FMT. This study suggested that the microbial abundance in the gut of FMT recipients was associated with the calf overall growth rate. These findings are in accordance with other studies that performed FMT in young livestock animals. For instance, Hu, et al. [52] orally inoculated newborn pigs from day 1 to day 11 of life with a fecal suspension from a healthy donor, and the recipient piglets had greater average daily gain than the control piglets. In addition, this study reported a lower incidence of diarrhea in the FMT group relative to control piglets [52]. Additionally, early life fecal inoculations from a highly feed-efficient donor chicken into broiler chickens tended to increase feed intake and BW in the FMT recipient’s chickens [53]. Therefore, our data add to the growing body of evidence that gut microbiota has a profound impact on the development of young animals. However, the biological mechanisms underlying the gut-host and FMT interactions that can impact the body mass (i.e., body conformation and feed intake) of calves remain to be elucidated.

### 4.1. Gut Microbial Maturation Impacted by FMT Early in Life

Several studies have shown that FMT and specific probiotics can shift gut microbiota towards a healthy state by reconstituting the symbiosis of the gastrointestinal tract [17,54,55]. In particular, FMT has been demonstrated to be an effective therapy for *Clostridioides difficile* (CD) infection, inflammatory bowel disease, and Crohn’s disease [19,20,23,24]. In addition, FMT has been reported to alleviate the inflammatory response in individuals experiencing other GIT disorders [56,57] as well as in cases of necrotizing enterocolitis via regulation of the oxidative stress response using mouse models [22,58]. In this study, the relative abundance of Firmicutes and Bacteroidetes across all calves was consistent with what has been reported as a healthy fecal microbiota composition of preweaned dairy calves [59]. Interestingly, the alpha diversity of the microbial communities increased from pre- (week 1) to post-FMT (week 5) in the feces of calves that received FMT. Similar improvements in alpha diversity in microbial communities have been observed in studies that performed FMT in beef growing calves [51] and infants diagnosed with recurrent CD infection [21]. Gut dysbiosis has been exemplified by an abnormal increase in the abundance of Enterobacteriaceae, which can trigger an inflammatory response [60]. In contrast, the gut of healthy adult subjects is composed of a minor proportion of this facultative anaerobic bacteria [61]. Recently, a longitudinal evaluation of the fecal microbial communities (from birth to 8 weeks of age) in dairy calves with diarrhea reported a greater abundance of *Enterobacteriaceae* in the feces of diarrheic calves relative to the control [62]. In the current study, the lower relative abundance of *Enterobacteriaceae* post-FMT in the feces of recipient’s calves at week 5 suggests that FMT may modulate gut microbial composition in early life. 

### 4.2. Neonatal Liver Function and Inflammation

In the neonatal liver, the synthesis of certain APPs can be related to the interaction of colostrum intake [63] and liver maturation [64] rather than a disease-related process, which makes the interpretation of APPs in neonates cumbersome. Liver maturation can be largely affected by bioactive compounds in colostrum, including cholesterol, hormones, and cortisol [65,66]. For instance, cortisol has been associated with liver maturation, which, in turn, can drive changes in energy metabolism in the neonate [67]. There is an evident change in hepatic metabolic activity from neonatal calves to the adult ruminants [68]. This change can be mainly attributed to alterations in the metabolizable substrates by the liver from pre-ruminant to ruminant (i.e., glucose and fatty acids vs. short-chain fatty acids [SCFA] due to rumen development). In this context, the shift from glucose intestinal absorption during the pre-weaning period to SCFA, ketones, and microbial protein absorption in an adult ruminant lead to changes in the hepatic function and energy metabolism during the transition from pre- to- ruminant, including the reduction in enzyme activity of glucose oxidation in the liver [68]. Then, the synthesis of hepatic proteins, including the negative APPs (e.g., paraoxonase) [69] in the neonatal stage, can be associated with liver maturation product, in part, by inductive factors in colostrum. The latter scenario may cause confounding, in terms of hepatic synthesis of APPs, between natural liver maturation and a disease-derived inflammatory condition in the neonatal calf [64,70,71]. 

The antioxidant and anti-inflammatory enzyme paraoxonase (PON) blood levels increased over time in healthy neonatal calves. In calves of 7 d of age average blood concentration of PON was 8.9 ± 5.2 U/mL, and in calves, with 28 d of age, the concentration was 42.8 ± 14.8 U/mL [70]. However, when calves demonstrated a degree of inflammation (i.e., diarrhea or respiratory disease), the average PON observed in the blood of sick calves was 3.7 ± 1.4 U/mL at 7 d of age and 24.5 ± 13.4 U/mL at 28 d of age. Thus, there is a significant difference in PON concentration in healthy vs. sick (i.e., inflammatory condition) neonatal calves. In the current study, greater PON was observed in FMT by three weeks of age, which suggests that liver function was not compromised by FMT, and further provides evidence for lack of detrimental effects of performing FMT in neonates. 

In the current study, the greater haptoglobin in FMT calves at 2 weeks of age confirms that fecal inoculations between 8 and 12 d of age challenged the host to some extent. This is in agreement with Bertoni, et al. [63], where they reported that haptoglobin greater than 0.2 g/L in blood of neonatal calves is associated with an inflammatory condition. However, the haptoglobin concentration in FMT calves was modest when compared with other studies, where dairy calves were challenged with an infection (Haptoglobin = 1.23 g/L) or suffer mild diarrhea (Haptoglobin = 0.35 g/L) [72,73]. 

### 4.3. FMT Effects on Inflammation and Oxidative Stress during Weaning

During the weaning period, the GIT of dairy calves undergoes anatomical and physiological changes, including the volume of the rumen in comparison to the other forestomachs, enhanced metabolism, altered energy substrates, and microbial shift [74,75]. Furthermore, the weaning period can impact the overall calf health by inducing an acute phase response accompanied by an increase in cortisol and the modulation of inflammatory cytokines [76]. In pigs, FMT treatment stimulated the adaptive immune development at weaning by increasing the plasma concentrations of cytokines produced by T helper cells [77]. Although the current study did not include any adaptive immune parameter, the decrease in the pro-inflammatory cytokines IL1-β and IL6 in blood of FMT calves during the weaning period (Table 4) suggests that FMT can attenuate the inflammatory response related with the weaning stress.

The balance between oxidants and antioxidants is the hallmark of oxidative stress. ROM and FRAP have been used as biomarkers for oxidant and antioxidant status, respectively, in adult animals and in neonatal calves [78,79]. Although no prior data is available on the influence of FMT in oxidative stress in dairy calves, a trial performed in a mice model for necrotizing enterocolitis suggested that FMT treatment can alleviate oxidative stress by decreasing intestinal eNOS activity through posttranslational modification via S-glutathionylation [22]. Results from our study indicate that dairy calves could benefit from an early life FMT procedure by reducing oxidative stress during the weaning period, as observed by the concomitant decline in ROM while maintaining FRAP concentrations. However, if this effect was related to decreased eNOS activity remains to be elucidated. 

### 4.4. PMNL Gene Expression

In general, the lack of effect in PMNL gene expression in the current study suggests that the FMT procedure did not trigger an activation of the immune system, at least the innate immune system. The *ZBP1* gene encodes the protein Z-DNA binding protein 1, which plays a role in sensing microbial pathogens, including viruses that are not recognized by *TLR2* or *TLR4* [80]. The *ZBP1* is a relatively novel gene, and its role in pathogen recognition was only elucidated less than two decades ago by Takaoka, et al. [81]. Therefore, there is limited information on the importance of *ZBP1* for dairy cattle, especially the neonatal calf. Overall, the upregulation of *ZBP1* expression could be associated with a transient inflammatory response triggered by colostrum intake and environmental pathogen exposure during the first week of life (Figure S3). The same effect was observed in neonatal calves born to dams regardless of prepartal dietary treatments [82]. However, the mRNA upregulation of *ZBP1* in FMT calves than CON remains unclear, especially at 1 week, since the same standard procedures were applied to all calves between birth and 1 week. The evident decline in *ZBP1* expression in FMT calves after 1 week could be partially attributed to the FMT inoculations from 8 to 12 days. There is evidence that bacterial pathogens such as the enteropathogenic *Escherichia coli* (EPEC) may introduce effectors genes into the intestinal enterocytes that will inhibit apoptosis and NFKB1 activation [80], and this has been extrapolated to ZBP1 inhibition [83]. Although there is no data on EPEC leading to a downregulation of *ZBP1* mRNA expression, it is conceivable that a pathogen or unknown mediator in the fecal material used in the FMT treatment may have led to the evident downregulation of *ZBP1* in FMT calves by modifying this pathogen recognition mechanism in the enterocytes without inducing diarrhea. 

Besides *ZBP1*, the lack of effect of FMT in any of the genes related to inflammation and cell receptors supports the notion that an early life FMT does not elicit a systemic inflammation. This was further sustained by the lack of clinical signs of diarrhea, fever, or loss of appetite. 

## 5. Conclusions

Overall, this study demonstrated that early life fecal microbiota transplantation into neonatal dairy calves is a relatively safe method and can influence calf growth and development. These data demonstrated that FMT performed using fecal material from a selected donor from the herd may cause a mild challenge to the calf during the neonatal period, but this does not appear to develop into a systemic inflammatory response accompanied by diarrhea. Early life FMT can modulate the gut microbial maturation in neonatal calves. At weaning, FMT treatment seemed to alleviate the negative stress of weaning by reducing the pro-inflammatory response and increasing the antioxidant potential. The mechanism by which fecal microbiota inoculation interacts with the host microbiota and regulates the immune response and GIT maturation in neonatal calves remains to be determined. The authors recognize the low sample size as a limitation of this study, and future studies with a larger statistical power will be needed to confirm the FMT effects detected in the current study. Additionally, optimization of a standard protocol for the selection of the donor cow needs further investigation, including selection of donors based on the genetics and reproductive traits of the herd. 

## Figures and Tables

**Table 1:** Growth, intakes, feed efficiency, and health parameters in Holstein dairy calves raised under a conventional nutritional program (CON) or subjected to fecal microbiota transplantation (FMT).

Parameter	Group		SEM ^2^	*p*-Value ^3^		
	CON	FMT		TRT	Week	T × W
**Growth parameters ^1^**						
BW, kg	50.6	52.8	0.91	0.10	<0.01	0.82
Birth BW, kg	41.6	38.8	2.52	0.43	--	--
Pre-weaning BW, kg	57.7	61.2	1.34	0.06	--	--
Final BW, kg	64.0	67.0	1.40	0.12	--	--
WH, cm	82.6	83.8	0.49	0.13	<0.01	0.34
Birth WH, cm	78.4	76.6	1.22	0.31	--	--
Pre-weaning WH, cm	85.2	86.4	0.64	0.21	--	--
Final WH, cm	85.9	87.9	0.61	0.03	--	--
ADG, kg/d	0.46	0.47	0.04	0.92	<0.01	0.99
**Intakes and feed efficiency**						
Stater intake, g/d	504	486	73	0.86	<0.01	0.26
Total intake, g/d	1027	1009	72	0.86	<0.01	0.23
G:F, g/g	0.40	0.40	0.03	0.96	<0.01	0.76
**Health parameters**						
Fecal scores ^4^	1.86	2.16	0.17	0.25	0.28	0.86
Scour days ^5^	3.3	5.5	2.1	0.48	--	--
Rectal temperature, °C	38.6	38.6	0.06	0.94	0.39	0.32

^1^ Growth parameters for body weight (BW) and wither height (WH) from 1 to 7 week of age, and at birth (0 week), pre-weaning (6 week), and final (7 week). ^2^ Largest standard error of the mean. ^3^ *p*-value for treatment (TRT), week, or interaction (TRT × week). ^4^ Fecal scores based on a 1 to 4 scale (1: well-formed-solid feces; 2: soft, pudding-like; 3: runny, pancake batter; 4: liquid, splatters). ^5^ Number of days with a fecal score of 4.

**Table 2:** Mean relative abundance (%) of the main bacterial groups identified in the fecal samples of control (CON) and fecal microbiota transplantation (FMT) calves, and the donor cow analyzed in this study.

OTU	Week 1		Week 2		Week 5		Donor
	CON	FMT	CON	FMT	CON	FMT	
**Firmicutes**	32.03	38.32	44.0	35.19	33.0	36.44	57.76
*Ruminococcaceae*	10.01	9.91	7.63	8.48	9.97	12.45	18.86
*Lachnospiraceae*	5.67	4.32	8.81	6.30	7.61	7.81	11.97
*Erysipelotrichaceae*	4.67	4.18	8.10	7.68	3.64	5.65	9.72
*Peptostreptococcaceae*	0.01	0.15	0.03	0.26	0.24	0.18	8.74
*Clostridiales Inc. Sedis XIII*	0.27	0.16	0.68	0.20	0.18	0.19	3.49
*Clostridiaceae1*	0.09	0.12	<0.01	0.06	0.85	0.05	1.46
*Lactobacillaceae*	1.03	1.73	2.15	0.58	1.50	0.49	0.14
*Streptococcaceae*	1.89	5.93	4.08	1.00	1.20	0.24	0.23
*Veillonellaceae* #	4.38	4.69	4.62	2.54	2.14	0.62	0
*Enterococcaceae* #	0.35	1.64	1.14	0.50	0.46	0.01	0
*Selenomonadaceae*	1.96	3.47	3.08	2.39	1.94	1.24	0.03
Other Firmicutes *	1.61	2.04	3.67	5.21	3.26	7.50	3.13
**Bacteroidetes**	30.01	24.94	34.70	34.27	35.22	33.29	31.25
*Muribaculaceae*	0.03	0.02	0.04	0.02	0.05	0.05	5.36
*Bacteroidaceae* #	5.48	0.56	5.96	2.72	2.09	1.62	4.79
*Odoribacteraceae* #	0.03 ^ab^	0.01 ^ab^	0.10 ^a^	<0.01 ^a^	0.20 ^bc^	0.18 ^c^	4.71
*Prevotellaceae*	22.95	23.68	24.42	30.41	30.35	28.08	3.63
*Porphyromonodaceae*	0.66	0.20	1.39	0.31	0.43	0.22	0.18
*Rikenellaceae*	0.03	0.04	0.07	0.08	0.02	0.04	3.05
Other Bacteroidetes *	0.81	0.43	2.73	0.71	2.08	3.11	9.52
**Proteobacteria**	28.73	29.95	12.98	24.98	21.96	22.53	6.72
*Succinivibrionaceae*#	2.32	2.81	0.85	8.84	6.20	4.13	4.27
*Pasteurellaceae Pasteurellaceae #*	2.69 ^ab^	2.18 ^ab^	1.82^a^	2.76 ^a^	2.52 ^ab^	0.04 ^b^	0
*Sutterellaceae*	12.49	9.17	5.32	8.39	9.23	8.26	0.20
*Enterobacteriaceae* #	10.61	14.59	3.63	2.70	2.21	0.79	0.20
Other Proteobacteria *	0.63	1.21	1.37	2.28	1.81	9.31	2.00
**Actinobacteria #**	2.27 ^ab^	2.27 ^a^	4.65^a^	1.17 ^ab^	2.08 ^ab^	0.75 ^b^	0.36
**Verrucomicrobia**	2.72	1.88	0.03	0.22	1.35	<0.01	0.01
**Campylobacterota**	3.74	2.31	2.37	1.33	2.06	0.06	0.01
Other Bacteria *	0.49	0.34	1.27	2.83	4.33	6.91	3.88

Abundance is presented as a percentage (%) of the total number of analyzed reads per sample. # OTUs that show a statistically significant difference (*p* < 0.05) across the control and FMT calves based on the Kruskal-Wallis rank sum test. This analysis did not include the donor because it was a single sample. ^abc^ Different superscripts in the same row indicate that groups are significantly different by the Wilcoxon pairwise test. * Statistical test not performed because of group heterogeneity.

**Table 3:** Blood immunometabolic biomarkers during the neonatal period (from birth to 3 weeks of age) in Holstein dairy calves raised under a conventional nutritional program (CON) or subjected to fecal microbiota transplantation (FMT).

Parameter ^1^	Group		SEM ^2^	*p*-Value ^3^		
	CON	FMT		TRT	Week	T × W
**Energy metabolism**						
Glucose, mmol/L	6.23	5.69	0.34	0.33	0.04	0.28
BHBA ^4^, mmol/L	0.09	0.07	0.01	0.20	0.36	0.28
Creatinine, µmol/L	94.65	91.75	4.41	0.65	0.03	0.72
Urea, mmol/L	2.98	2.74	0.16	0.29	0.01	0.07
**Inflammation**						
Haptoglobin, g/L	0.19	0.21	0.01	0.24	0.06	0.02
Ceruloplasmin, µmol/L	1.82	1.95	0.13	0.51	0.38	0.29
IL1-β (log2) pg/mL	3.69	3.67	0.39	0.98	0.26	0.07
IL-6, (log2) pg/mL	8.17	7.96	0.20	0.48	<0.01	0.68
**Liver function**						
Albumin, g/L	30.61	30.03	0.29	0.18	<0.01	0.14
Paraoxonase, U/mL	34.50	40.56	2.19	0.07	<0.01	0.06
Total bilirubin, µmol/L	4.67	6.30	0.97	0.26	<0.01	0.13
Cholesterol, mmol/L	2.68	2.40	0.17	0.25	<0.01	0.31
GOT ^5^ (log2), U/L	5.76	5.89	0.11	0.42	<0.01	0.71
GGT ^6^, U/L	458.4	418.1	87.4	0.76	<0.01	0.16
**Oxidative Stress**						
ROM ^7^, mg H2O2/100 mL	12.06	12.09	0.60	0.98	0.23	0.20
FRAP ^8^, mmol/L	160.45	149.64	7.79	0.36	0.46	0.34

^1^ Metabolites and biomarkers were analyzed from 1 to 3 weeks of age. ^2^ Largest standard error of the mean is shown. ^3^ *p*-value for treatment (TRT), week, or interaction (TRT × week). ^4^ BHBA = β-hydroxybutyric acid. ^5^ GOT = glutamic-oxaloacetic transaminase. ^6^ GGT = γ-glutamyltransferase. ^7^ ROM = reactive oxygen metabolites. ^8^ FRAP = ferric reducing antioxidant power.

**Table 4:** Blood immunometabolic biomarkers during the weaning period (6- to 7-week old) in Holstein dairy calves raised under a conventional nutritional program (CON) or subjected to fecal microbiota transplantation (FMT).

Parameter ^1^	Group				SEM ^2^	*p*-Value ^3^		
	CON		FMT					
	6	7	6	7		TRT	Week	T × W
**Energy metabolism**								
Glucose, mmol/L	5.61	4.93	5.63	5.12	0.25	0.78	<0.01	0.34
BHBA ^4^, mmol/L	0.18	0.25	0.15	0.26	0.03	0.86	0.02	0.62
Creatinine, µmol/L	80.9	82.3	80.9	82.4	3.5	0.99	0.45	0.98
Urea, mmol/L	4.43	7.29	3.68	6.0	0.34	0.05	<0.01	0.33
**Inflammation**								
Haptoglobin (log2), g/L	0.22	0.47	0.32	0.49	0.26	0.48	0.05	0.54
Ceruloplasmin, µmol/L	1.50	1.72	1.62	1.69	0.19	0.88	0.14	0.40
IL1-β (log2) pg/mL	27.5	12.0	11.6	4.6	0.27	0.01	0.01	0.87
IL-6, (log2) pg/mL	159.2	154.4	72.2	69.0	0.36	0.05	0.87	0.98
**Liver function**								
Albumin, g/L	32.4	33.2	32.7	33.3	0.50	0.83	0.09	0.84
Paraoxonase, U/mL	77.5	74.0	92.8	85.3	5.85	0.09	0.18	0.61
Total bilirubin, µmol/L	2.49	2.36	3.12	2.78	0.40	0.38	0.41	0.72
Cholesterol, mmol/L	3.66	2.69	3.89	3.08	0.24	0.37	<0.01	0.69
GOT ^5^ (log2), U/L	102.4 ^a^	87.1 ^b^	82.4 ^b^	89.2 ^ab^	0.11	0.33	0.37	0.02
GGT ^6^ (log2), U/L	84.6 ^a^	43.7 ^bc^	44.5 ^b^	30.0 ^c^	0.29	0.07	<0.01	0.01
**Oxidative Stress**								
ROM ^7^, mg H2O2/100 mL	10.2 ^b^	13.1 ^a^	10.2 ^a^	9.6 ^b^	1.11	0.25	0.07	0.01
FRAP ^8^, mmol/L	159.8 ^a^	137.7 ^b^	161.5 ^a^	161.0 ^a^	9.3	0.30	0.01	0.01

^1^ Metabolites and biomarkers were analyzed at 6 and 7 weeks of age, and log-transformed variables were back-Table 2. ^2^ Largest standard error of the mean is shown, and SEM of log-transformed variables remained in log-scale. ^3^ *p*-value for treatment (TRT), week, or interaction (TRT × week). ^4^ BHBA = β-hydroxybutyric acid. ^5^ GOT = glutamic-oxaloacetic transaminase. ^6^ GGT = γ-glutamyltransferase. ^7^ ROM = reactive oxygen metabolites. ^8^ FRAP = ferric reducing antioxidant power. ^abc^ abSuperscript letters indicate significant (*p*-value < 0.05) difference between means in the same row.

**Table 5:** Gene expression in polymorphonuclear leukocytes (PMNL) during the neonatal period (from birth to 3 week of age) in Holstein dairy calves raised under a conventional nutritional program (CON) or subjected to fecal microbiota transplantation (FMT).

Transcript ^1^	Group		SEM ^2^	*p*-Value ^3^		
	CON	FMT		TRT	Week	T × W
*ZBP1*	1.09	1.85	0.23	0.03	0.40	0.64
*NFKB1*	1.21	1.57	0.15	0.12	0.20	0.53
*STAT3*	−0.50	−0.34	0.21	0.60	0.87	0.92
*SELL*	0.82	1.27	0.30	0.30	0.10	0.86
*TLR2*	1.24	1.41	0.27	0.66	0.50	0.11
*TLR4*	0.40	0.65	0.26	0.50	0.36	0.36
*IL1B*	0.62	0.74	0.40	0.84	0.37	0.45
*IL8*	0.27	0.87	0.54	0.44	0.01	0.60

^1^*ZBP1* = Z-DNA binding protein 1, *NFKB1* = Nuclear factor kappa B subunit 1, *STAT3* = Signal transducer and activator of transcription 3, *SELL* = Selectin L, *TLR2* = Toll-like receptor 2, *TLR4* = Toll-like receptor 4, *IL1B* = Interleukin 1 beta, *IL8* = Interleukin 8. ^2^ Largest standard error of the mean. ^3^ *p*-value for treatment (TRT), week, or interaction (TRT × week).

## Data Availability

Raw sequence data are available in NCBI Sequence Read Archive under Bioproject PRJNA762384 and SRA accession SRP336636.

## Supplementary Materials

The following are available online at https://www.mdpi.com/article/10.3390/ani11092704/s1, Table S1: General information of potential donor cows, including lactation number, average 305 mature equivalent, days in milk, and results for analysis of infectious pathogens. Table S2: Relative abundance of the most highly represented fecal bacterial OTUs from the fecal donor used in this study. Abundance is presented as a percentage (%) of the total number of non-chimeric reads per sample. Table S3: Closest valid relatives for the main bacterial fecal OTUs identified in this study. Figure S1: Rectal temperature (°C) after a fecal microbiota transplantation (FMT) within antibiotic-free milk replacer into neonatal Holstein calves during a pre-trial phase of 4 days. Figure S2: Principal Coordinates Analysis (PCoA) of the fecal microbial community identified across samples of the control calves, fecal microbiota transplantation recipient’s calves (Transplant), and the donor cow at week 1, week 2, and week 5. Figure S3: Relative mRNA expression of ZBP1 gene in polymorphonuclear leukocytes (PMNL) of Holstein dairy calves raised under a conventional nutritional program (CON) or subjected to fecal microbiota transplantation (FMT). The *p*-values for main effect of treatment (TRT), and week and TRT × Week are shown. Data before week 1 of age are from samples taken 24 h after birth (baseline).
