# Supplementary material for: Early Life Fecal Microbiota Transplantation in Neonatal Dairy Calves Promotes Growth Performance and Alleviates Inflammation and Oxidative Stress during Weaning
Source: Animals (Basel). 2021 Sep 15;11(9):2704. doi: 10.3390/ani11092704 (PMC8471931; doi:10.3390/ani11092704)
Supplement: Supplementary file 1 [file animals-11-02704-s001.zip › Supplementary Files.pdf]

Table S1. General information of potential donor cows, including lactation number, average 305 mature equivalent, days in milk, and results for analysis of infectious pathogens.

Cow	Lactation	Average 305 ME	DIM[1]	Salmonella	Aerobic culture	Cryptosporidium	Fecal float for ova	Mycobacterium paratuberculosis
7106	5	11,131	74	-	-	+	-	-
7183	3	11,314	245	-	-	+	+	-
7186[2]	5	12,772	122	-	-	-	-	-
7125	4	10,521	82	-	-	-	-	-
7044	5	10,866	38	-	-	+	-	-

[1]Days in milk at the time of fecal sample collections.
[2]Selected cow for FMT procedure in neonatal calves.

Table S2. Relative abundance of the most highly represented fecal bacterial OTUs from the fecal donor used in this study. Abundance is presented as a percentage (%) of the total number of non-chimeric reads per sample.

**OTU**	**%**	**Closest valid relative$ (% id.)**	**Closest uncultured# (% id.)**
Bt-01095	7.40	Turicibacter sanguinis (98.8%)	KJ854177.1 (100%)
Bt-01143	5.26	Blautia faecicola (97.1%)	GQ898319.1 (100%)
Bt-01320	4.44	Duncaniella dubosii (85.7%)	FJ681443.1 (99.4%)
Bt-01111	4.34	Romboutsia timonensis (99.2%)	JQ307275.1 (99.8%)
Bt-01112	3.30	Succinivibrio dextrinosolvens (96.9%)	GQ448632.1 (100%)
Bt-19378	2.93	Romboutsia lituseburensis (96.7%)	HG964582.1 (99.8%)
Bt-65419	2.31	Duncaniella dubosii (90.4%)	EU775229.1 (92.4%)
Bt-65420	2.28	Millionella massiliensis (82.6)	EU474821.1 (98.9%)
Bt-01001	1.90	Roseburia inulinivorans (95.1%)	EU475442.1 (97.9%)
Bt-65445	1.79	Devosia marina (82.6%)	AB555253.1 (86.8%)
Bt-01013	1.61	Oscillibacter valericigenes (86.8%)	FJ683233.1 (99.4%)
Bt-01141	1.50	Prevotella oulorum (90.7)	JN884094.1 (99.6%)
Bt-65468	1.36	Bacteroides caecigallinarum (88.8)	GQ448718.1 (98.9%)
Bt-01078	1.32	Clostridium saudiense (99.0)	GU120126.1 (100%)
Bt-01070	1.31	Prevotella copri (99.2)	FJ683633.1 (99.8%)
Bt-65475	1.26	Duncaniella freteri (87.0)	EU468645.1 (99.8%)
Bt-19397	1.23	Casaltella massiliensis (89.1)	EU777315.1 (99.8%)
Bt-65498	1.04	Millionella massiliensis (82.6)	GU603694.1 (99.8%)

$Sequence from the NCBI 'refseq_rna' database with the highest nucleotide sequence identity as determined by BLAST.
#Sequence from the NCBI 'nt' database with the highest nucleotide sequence identity as determined by BLAST.

Table S3. Closest valid relatives for the main bacterial fecal OTUs identified in this study.

OTU	Closest valid taxon (%id)*	Taxonomic affiliation
Bt-01001	*Clostridium bolteae* (96.64%)	Firmicutes
Bt-01021	*Streptococcus macedonicus* (99.25%)	Firmicutes
Bt-01063	*Shigella sonnei* (99.81%)	Proteobacteria
Bt-01065	*Bacteroides vulgatus* (99.62%)	Firmicutes
Bt-01070	*Prevotella copri* (99.24%)	Bacteroidetes
Bt-01078	*Clostridium saudiense* (99.0%)	Firmicutes
Bt-01095	*Turicibacter sanguinis* (98.81%)	Firmicutes
Bt-01111	*Romboutsia timonensis* (99.18%)	Firmicutes
Bt-01112	*Succinivibrio dextrinosolvens* (96.88%)	Proteobacteria
Bt-01141	*Prevotella oulorum* (90.72%)	Bacteroidetes
Bt-01142	*Gloeobacter kilaueensis* (82.23%)	Proteobacteria
Bt-01143	*Blautia faecicola* (97.09%)	Firmicutes
Bt-01163	*Megasphaera elsdenii* (99.82%)	Firmicutes
Bt-01193	*Muribaculum intestinale* (89.39%)	Bacteroidetes
Bt-01201	*Collinsella aerofaciens* (98.38%)	Actinobacteria
Bt-01205	*Prevotellamassilia timonensis* (89.64%)	Bacteroidetes
Bt-01207	*Blautia luti* (97.81%)	Firmicutes
Bt-01208	*Prevotella stercorea* (99.05%)	Bacteroidetes
Bt-01214	*Prevotella amnii* (90.87%)	Bacteroidetes
Bt-01230	*Sutterella massiliensis* (99.62%)	Proteobacteria
Bt-01238	*Gallibacterium anatis* (96.39%)	Proteobacteria
Bt-01351	*Kiloniella majae* (85.27%)	Proteobacteria
Bt-01356	*Anaeromassilibacillus senegalensis* (83.53%)	Firmicutes
Bt-01366	*Succiniclasticum ruminis* (87.70%)	Firmicutes
Bt-01391	*Sharpea azabuensis* (100%)	Firmicutes
Bt-01402	*Faecalibacterium prausnitzii* (97.78%)	Firmicutes
Bt-01403	*Succinivibrio dextrinosolvens* (92.92%)	Proteobacteria
Bt-01406	*Acidaminococcus fermentans* (99.63%)	Firmicutes
Bt-01409	*Succinivibrio dextrinosolvens* (97.52%)	Proteobacteria

* Percentage of identical nucleotides between each OTU and its corresponding closest valid relative.

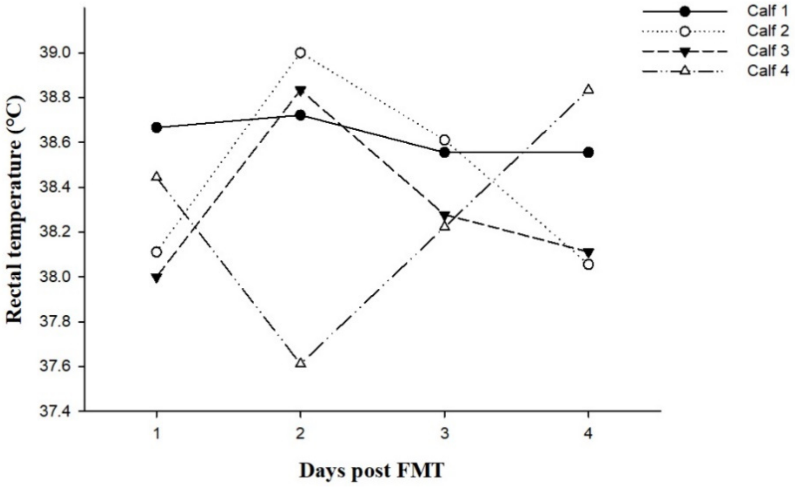

Figure S1. Rectal temperature (°C) after a fecal microbiota transplantation (FMT) within antibiotic-free milk replacer into neonatal Holstein calves during a pre-trial phase of 4 days.

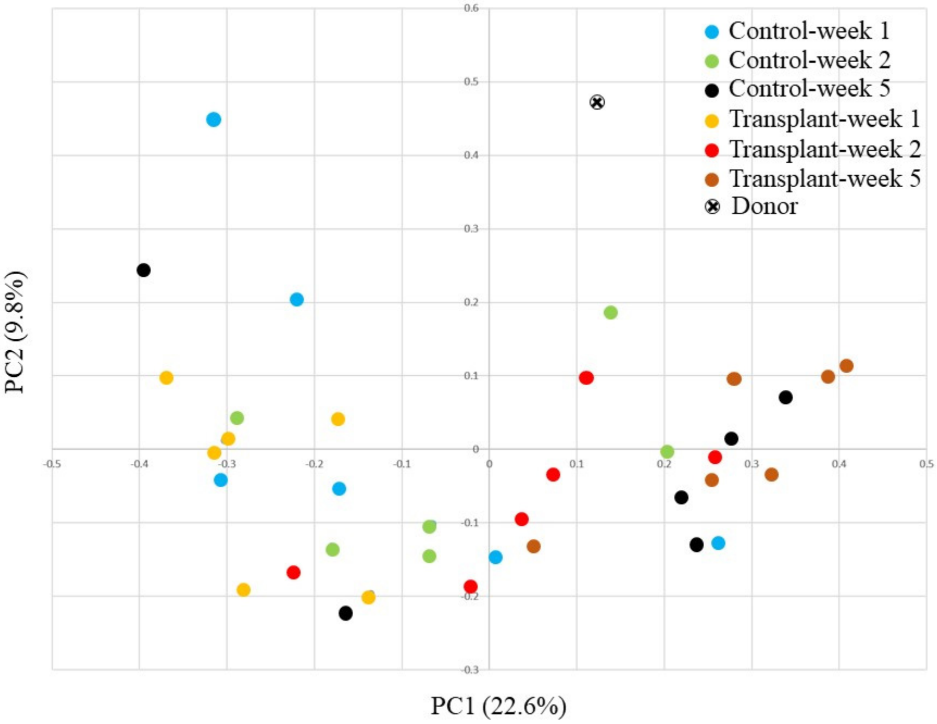

Figure S2. Principal Coordinates Analysis (PCoA) of the fecal microbial community identified across samples of the control calves, fecal microbiota transplantation recipient's calves (Transplant), and the donor cow at week 1, week 2, and week 5.

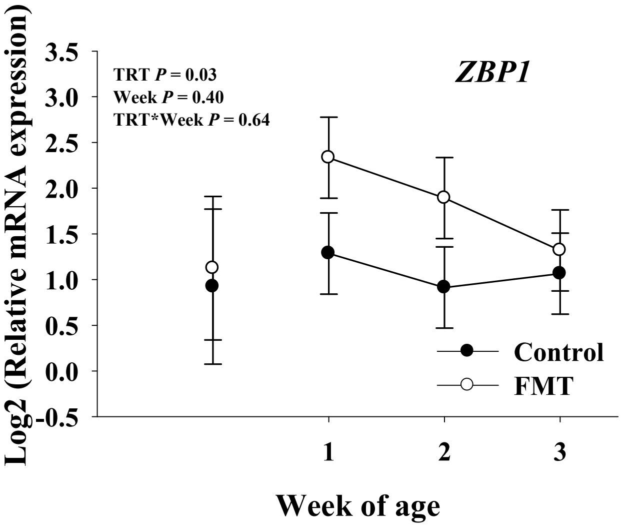

Figure S3. Relative mRNA expression of ZBP1 gene in polymorphonuclear leukocytes (PMNL) of Holstein dairy calves raised under a conventional nutritional program (CON) or subjected to fecal microbiota transplantation (FMT). The P-values for main effect of treatment (TRT), and week and TRT×Week are shown. Data before week 1 of age are from samples taken 24 h after birth (baseline).
